# Palladium Porphyrin-Modified Fibrin Hydrogel for Oxygen Sensing in Engineered Skin Tissue Applications

**DOI:** 10.1021/acsomega.6c03389

**Published:** 2026-05-13

**Authors:** Ana Matesanz, Marta Ruiz-Llata, Raúl Sanz-Horta, Paula Bosch, Carlos Elvira, Helmut Reinecke, Jose Luis Jorcano, Pablo Acedo

**Affiliations:** † Department of Electronic Technology, 16726 Universidad Carlos III de Madrid (UC3M), Madrid 28911, Spain; ‡ Department of Bioengineering, Universidad Carlos III de Madrid (UC3M), Madrid 28911, Spain; § Department of Applied Macromolecular Chemistry, Institute of Polymer Science and Technology, 83077 Spanish National Research Council (ICTP-CSIC), Juan de la Cierva 3, Madrid 28006, Spain; ∥ Instituto de Investigación Sanitaria Gregorio Marañón, Madrid 28007, Spain

## Abstract

Tissue engineering is transforming regenerative medicine by enabling the creation of functional tissue substitutes for in vitro research and therapeutic applications. Optimization of skin-engineered tissues would clearly be favored by local, real-time, and precise oxygen concentration measurements, as oxygen plays a fundamental role in cellular metabolism, survival, and tissue regeneration. This work presents a fluorescence-based oxygen biosensor that is chemically stable and integrated into fibrin-based dermal matrices used as scaffolds in complete skin equivalents (including dermis and epidermis). The process involves synthesizing PdPFP-Jeff, a modified porphyrin incorporating palladium and Jeffamine ED 600, and covalently bonding it to alginate modified with *N*-hydroxysuccinimide (AlgNHS), previously introduced within plasma-derived hydrogels that would form the skin construct in vitro. The chemical bonding enables stable incorporation, preventing leaching and maintaining structural integrity, while also ensuring biocompatibility with aqueous environments. Various biological assays, including live/dead assays, hematoxylin and eosin staining, and immunofluorescence studies, were conducted to confirm the biocompatibility of PdPFP-Jeff-enhanced dermoepidermal equivalents. When applied to hydrogels containing primary fibroblasts (dermal equivalents), this technique produced reliable results for these physiologically significant systems and demonstrated the potential of PdPFP-Jeff hydrogels as biosensors for real-time oxygen monitoring. By integrating high-resolution optical sensing into skin equivalents, this approach advances smart biomaterials, and improves in vitro skin models, and paves the way for enhanced wound healing and regenerative medicine applications.

## Graphical abstract

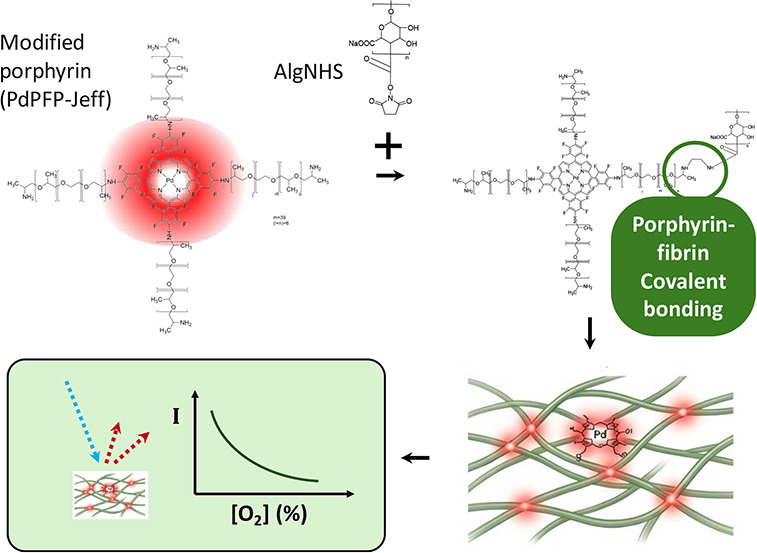

## Introduction

Tissue engineering is a multidisciplinary field that integrates biological sciences, engineering principles, and materials science to develop biological substitutes that restore, maintain, or enhance tissue function.^1,2^ This field plays a crucial role in regenerative medicine, particularly in the development of engineered skin constructs for wound healing and tissue regeneration. These constructs aim to replicate the structural and functional complexity of native skin, thereby improving the healing process and minimizing complications such as infections and chronic wound formation. However, the success of tissue engineering depends on a thorough understanding of tissue functionality and the ability to monitor dynamic processes occurring within engineered structures. In this sense, there is currently an effort to design functional tissues endowed with sensory capabilities, providing real-time insights into tissue state and functionality.^3^ Embedded sensors enable continuous monitoring and early detection of changes, like insufficient tissue oxygenation or inflammation, enhancing the understanding of tissue behavior and improving cell-based therapies.^4^ Eventually, sensory functions could be accompanied by actuator functions, opening new possibilities for dynamic and responsive tissue structures.^5–7^

One of the key challenges in tissue engineering is the precise and noninvasive measurement of oxygen concentration in the outer and deep layers of the tissue, as oxygen plays a vital role in cellular metabolism, angiogenesis, infection control, and overall tissue viability.^8^ Skin is an ideal model for studying oxygen dynamics due to its accessibility and its direct implication in wound healing, ischemia, and hypoxia-related disorders, where oxygen imbalance can impair tissue regeneration.^9,10^ Adequate oxygen levels are essential for promoting normal cellular functions, facilitating vascularization, and preventing bacterial proliferation, ultimately supporting the formation of functional skin substitutes.^11^ Numerous methods have been reported for tissue oxygen content measurement that can be categorized into invasive and noninvasive techniques.^12,13^ While invasive methods provide precise data, they carry risks such as tissue damage or infection.^14^ Noninvasive methods, though safer, are limited by their penetration depth and typically indirect nature.^15–17^

This work focuses on developing and validating a noninvasive, real-time, high-spatial-resolution method for measuring oxygen concentration in engineered skin tissues. The proposed method is based on the use of fluorescent porphyrins, which are oxygen-sensitive biomolecules whose fluorescence intensity and lifetime vary depending on oxygen concentration, making them valuable tools for biosensing and imaging applications.^18,19^ The use of porphyrins, externally injected or introduced by surgeries into the tissues, has already been reported for tissue oxygen concentration and oxygen partial pressure measurements.^20–22^ However, these approaches are fundamentally invasive and can provide information only about the oxygen concentration in the outer layer of the tissue due to poor penetration.

In contrast to previous proposals, in this work, we present a new approach based on chemically bonding the porphyrins to the tissue extracellular matrix (in our case, a hydrogel) to provide stable sensory centers embedded into the tissue. To that aim, this study introduces a novel method using modified palladium porphyrins (PdPFPs). These are functionalized via PEGylation with Jeffamines, a linear molecule containing primary amino groups attached to the end of a backbone that contains polyethylene glycol (PEG) and polypropylene glycol (PPG) in its structure to create a new compound we call PdPFP-Jeff. This functionalization enhances the biocompatibility and stability of the porphyrins,^23,24^ which will be directly integrated into the hydrogel, acting as an extracellular matrix in functional *in vitro*-engineered skin tissues. This is possible because the resulting PdPFP-Jeff compound forms stable amide chemical bonds with *N*-hydroxysuccinimide (NHS) functionalized hydrogels,^25^ enabling real-time oxygen sensing in engineered tissues associated with the fluorescence properties of the porphyrins discussed above. The developed system not only could provide valuable information on the effects of oxygen on cellular health but also represents a practical and scalable tool for assessing oxygenation in biological systems, including engineered skin equivalents and other tissue models. Beyond enhancing our understanding of oxygen dynamics in engineered tissues, this work contributes to the broader field of regenerative medicine, paving the way for improved therapeutic strategies in wound healing and tissue-engineering applications.

## Experimental Section

### Synthesis of Modified Porphyrin (PdPFP-Jeff)

The objective of the chemical modification of porphyrin by PEGylation was to produce a novel compound, the PdPFP-Jeff, capable of forming covalent chemical bonds with amines. PEGylation introduced solubilizing groups to the porphyrins, preventing aggregation in water and facilitating amine attachment to NHS (*N*-hydroxysuccinimide) groups. The metalloporphyrin used was a perfluorinated palladium porphyrin (PdPFP), in which the fluorine atom in the *para* position of the aromatic rings can be substituted by nucleophilic addition. The Jeffamine used (Jeff) was a polyether diamine with an average molecular weight of 600 Da and a predominantly poly­(ethylene oxide) backbone. Using microwave irradiation, up to four Jeffamine chains can be bonded to the porphyrin’s external rings by selective displacement of para-fluorine groups.^26^ Specifically, in this work, 13 mg of palladium porphyrin (PdPFP) were dissolved in 1 mL of NMP (*N*-Methyl-2-pyrrolidone). An excess of 230 mg of Jeffamine ED-600 (1:40 molar ratio PdPFP:Jeffamine) was added, and the mixture was stirred for 5 min. The solution was placed in a microwave reaction vial G10 (10 mL), subjected to microwave irradiation at 150 °C for 7 min at 600 rpm using an Anton Paar Monowave 300 (AP-MW) reactor, followed by a cooling period to 55 °C.

To confirm the success of the reaction, we used thin-layer chromatography (TLC). The reaction mixture was analyzed by TLC in order to confirm PEGylation, using silica gel plates (TLC Silica Gel 60G F254, 100390, Merck) and a 9:1 ratio of dichloromethane to methanol (CH_2_Cl_2_:CH_3_OH) solvent system. The plates were visualized under UV light to confirm the presence of PdPFP-Jeff.

Because the solution obtained via microwave irradiation (PdPFP-Jeff) contains an excess of free Jeffamine ED-600, dialysis was employed to remove it. Dialysis was performed using RC prewetted tubing Spectra/Por^TM^ 6 membranes with a 1 kDa MWCO (molecular weight cutoff). The reaction mixture (2 mL) was dialyzed against 500 mL of solvent and covered with aluminum foil, with solvent changes twice a day for 15 days: 12 days with NaCl 0.1% (pH 7.5) and 3 days with distilled water (pH 7.5). The purified PdPFP-Jeff was stored at −20 °C.

### Preparation of Oxygen-Sensing Hydrogels

The purified and modified PdPFP-Jeff porphyrin was subsequently incorporated into hydrogels composed of alginate modified with *N*-hydroxysuccinimide (AlgNHS) polymeric complexes. We obtained blood plasma-derived fibrin-modified alginate hydrogels that are biocompatible, support cell growth, and can incorporate our modified porphyrin (PdPFP-Jeff) due to their hydrophilic nature and NHS presence. These properties make AlgNHS hydrogels suitable for various biomedical applications, including tissue engineering, drug delivery, and biomolecule inclusion, as reported in the literature.^27,28^

PdPFP-Jeff was incorporated in the polymerization of AlgNHS hydrogels by reacting its Jeffamine-modified amines with the NHS moieties, which act as reactive groups that facilitate the formation of stable amide-type bonds. The synthesis and polymerization with fibrin from human plasma of AlgNHS hydrogels were performed following the process and methods described by Matesanz A. et al.^25^ and Montero A. et al.^27^ The first step was to dissolve the synthesized AlgNHS polymer in 0.9% NaCl (w/v) at 37 °C to achieve final AlgNHS concentrations of 0.5 and 1 mg/mL. Platelet-poor plasma (PPP) was then added to achieve a final fibrin concentration of 1.2 mg/mL (0.12% w/v). The NaCl-AlgNHS-fibrin solution was incubated at 37 °C, 5% CO_2_, and 70% relative humidity, in a cellular incubator (SCO_2_W) (Shel Lab CO_2_, Cascade Technical Sciences, Inc.) for 20 min to allow amination between AlgNHS and plasma proteins. Next, the NaCl/AlgNHS/fibrin solution is combined with the synthesized, dried, and purified PdPFP-Jeff at 0.01%, 0.05%, or 0.1% (w/v) for an additional 20 min under the same conditions in order to form stable amide bonds between the primary amines in the PdPFP-Jeff and the remaining NHS ester groups in the AlgNHS. Hydrogel polymerization was initiated by adding 0.08% (w/v) CaCl_2_ (prepared as a 1% w/v solution in 0.9% NaCl) and incubating for 1 h under the same conditions.

### Viability of Cell Cultures in Oxygen-Sensing Hydrogels

Human primary fibroblasts (hFBs) and keratinocytes (hKCs) were obtained from PromoCell (Germany). Fibroblasts were cultured in Dulbecco’s Modified Eagle’s Medium (DMEM, without phenol red) with 10% Fetal Bovine Serum (FBS) and 1% Penicillin/Streptomycin (P/S) at 37 °C, 5% CO2. Keratinocytes were cultured basically according to the seminal method proposed by Rheinwatd J. G. and Green.^29^ Irradiated mouse embryonic fibroblasts (3T3-J2) were used to create the feeder layer necessary for keratinocyte growth. The culture medium used was DMEM/Ham-F12 (3:1), enhanced with 10% Fetal Calf Serum (FCS), 5 μg/mL of insulin, 1.3 ng/mL triiodothyronine, 8 ng/mL cholera toxin, 24 μg/mL adenine, 0.4 μg/mL hydrocortisone, and 5 μg/mL of insulin, along with 1% P/S and 10 ng/mL of EGF (Epidermal Growth Factor). For cell viability analysis (Live/Dead assays, Thermo Fisher Scientific), hydrogels with hFBs or hKCs were incubated with Calcein AM (0.5 μL/mL) and Ethidium Homodimer-1 (1 μL/mL) for 40 min at 37 °C and washed with Phosphate-Buffered Saline (PBS). Under these conditions, green and red cells represent viable and dead cells, respectively. For hFBs, 160,000 cells/mL of hydrogel were embedded inside the hydrogels before polymerization and analyzed after 7 days of culture. hKCs were seeded on the top of the hydrogels in a volume of 200 μL of culture medium CnT-57 (7,000 cells/well, or approximately 20,600 cells/cm^2^)

Hydrogels were used at final concentrations of 0, 0.01, 0.05, and 0.1% (w/v) at different AlgNHS concentrations (0.5 and 1 mg/mL) and were analyzed using a confocal microscope, Leica-SPE (Leica, Germany). Experiments were repeated at least three times.

### Three-D Dermo-Epidermal Equivalents in Oxygen-Sensing Hydrogels

To form dermal and dermo-epidermal 3-D equivalents, hFBs (20,000 cells/mL) were added to the fibrin-containing hydrogel mixture (PdPFP-Jeff) before hydrogel polymerization, and hKCs (240,000 cells/cm^2^) were seeded on top after hydrogel polymerization. Based on the results obtained during the course of the experiments performed (see the Experimental Section), PdPFP-Jeff hydrogels containing AlgNHS at a final concentration of 0.5 mg/mL and PdPFP-Jeff at final concentrations of 0.01% and 0.05% (w/v) were used to generate dermo-epidermal matrices. The method described by Montero A. et al.^27^ and Meana A. et al.^30^ was used to prepare dermo-epidermal matrices *in vitro*. After the 15-day incubation period at the air–liquid interface, required for epidermal terminal differentiation, the skin equivalents were analyzed by Hematoxylin & Eosin (H&E) staining and immunofluorescence studies. For H&E staining, they were fixed, cut into 5 μm slices, and stained according to Meana A. et al.^30^ Terminal epidermal differentiation was analyzed by immunofluorescence using antibodies against the following markers: Involucrin (Involucrin Monoclonal Antibody (MA5–11803), Thermo Fisher Scientific, USA) for the identification of the suprabasal spinous layer; Loricrin (Loricrin Polyclonal Antibody (PA5–30583), Thermo Fisher Scientific, USA), which stains the epidermal granular layer; Cytokeratin 5 (Cytokeratin 5 Monoclonal Antibody (PA5–32465), Thermo Fisher Scientific, USA) for the identification of basal human keratinocytes and Cytokeratin 10 (Cytokeratin 10 Monoclonal Antibody (MA5–13705), Thermo Fisher Scientific, USA) for the labeling of suprabasal keratinocytes. For the detection of nuclei in the dermo-epidermal matrices, DAPI staining (Thermo Fisher Scientific, USA) was used. Three experiments of this type were performed and analyzed for each experimental condition.

### Characterization Equipment

We performed MALDI-TOF (matrix-assisted laser desorption/ionization time-of-flight) analysis before and after dialysis for the quantification of PdPFP-Jeff and the remaining unmodified PdPFP. Samples were mixed with trans-2-[3-(4-*tert*-butylphenyl)-2-methyl-2-propenylidene] malononitrile (DCTB) matrix and analyzed using a Bruker Ultraflex mass spectrometer.

We performed spectral absorption analysis to confirm the stable chemical bonding of porphyrin to the polymer (the PdPFP-Jeff reaction with the NHS groups in AlgNHS). A Synergy HTX Multi-Mode Microplate Reader (Winooski, USA), with a wavelength range of 200–800 nm, was used.

We performed a biocompatibility analysis of oxygen-sensing hydrogels (PdPFP-Jeff plasma-derived fibrin hydrogels). Cell viability was assessed on days 3 and 7 of culture using Live/Dead assays (Thermo Fisher Scientific). The fluorescence was analyzed using a Leica-SPE (Leica, Germany) confocal microscope.

The significance of our investigation lies in the ability to accurately measure the phosphorescence of our oxygen-sensing hydrogels under varying oxygen concentrations. To that aim, we used a PerkinElmer LS 55 fluorescence spectrometer to obtain fluorescence intensity measurements. We selected an excitation wavelength of 405 nm to minimize porphyrin photobleaching and reduce autofluorescence interference.^31^ The emission spectra of the hydrogels were recorded (from 450 to 750 nm with 0.5 nm steps) at room temperature under varying oxygen concentrations, and the intensity fluorescence data were corrected for the background signal of the spectrometer. To perform the measurements, a volume of 700 μL of oxygen-sensing hydrogel (with and without cells) was placed in HELLMA Macro-101-QS cuvettes. Next, the cuvettes were sealed using rubber stopers, and two standard hypodermic needles along with sterile rubber tubes were inserted to allow gas exchange. A black blanket was used to cover the entire setup. Nitrogen injection was used to deplete the oxygen concentration inside sealed chambers containing the hydrogels, and the intensity changes were measured over time. An oxygen concentration of 0% was identified by the fluorescence intensity reaching a plateau during ten consecutive identical measurements after nitrogen was injected to deplete the oxygen. After that, equilibrium with the atmospheric oxygen concentration was reached (21%) by opening the chamber containing the hydrogel. An oxygen concentration of 21% was also identified by the fluorescence intensity reaching a plateau during ten consecutive identical measurements after the chamber was opened. The process was repeated three times per hydrogel sample. To assess and compare the sensitivity of the fluorescence signal to oxygen concentration, we used the fluorescence enhancement factor (FeF) at the maximum emission wavelength (λ_em_ = 672 nm). To calculate this parameter, we previously characterized the autofluorescence of the hydrogel by measuring the fluorescence intensity of a control hydrogel without PdPFP-Jeff. The FeF was obtained as:

1 $$FeF=\frac{I_{0\%}-I_{0\%,\mathrm{control}}}{I_{21\%}-I_{21\%,\mathrm{control}}}$$

where *I*_*0%*_ and *I*_*21%*_ are the values of the intensity peaks of the PdPFP-Jeff hydrogel sample for oxygen concentrations of 0% and 21%, respectively, and *I*_*0%,control*_ and *I*_*21%,control*_ are the values of the intensity peaks of the control hydrogel sample for oxygen concentrations of 0% and 21%, respectively.

## Results and Discussion

### Efficiency of the Synthesis Process of Modified Porphyrin (PdPFP-Jeff)

The characterization of the dialyzed PdPFP-Jeff involved analyzing and quantifying the concentration of free Jeffamines in the polymer solution. The final product contained pure palladium metalloporphyrin, PdPFP-Jeff with 1, 2, 3, and 4 bound Jeffamines, and pure Jeffamine (free Jeffamine). As observed in Figure 1, before dialysis, the MALDI-TOF analysis revealed predominant peaks around 600–650 *m*/*z* associated with free Jeffamine ED-600, with this Jeffamine displaying multiple oligomeric peaks at higher *m*/*z* values due to its polymeric nature.^32^ Peaks at 1094 *m*/*z* for porphyrin without modification and peaks for PdPFP-Jeff can also be observed with lower relative intensity. After dialysis, multiple additional peaks can be identified, corresponding to PdPFP-Jeff at approximately 1,600, 2,200, 2,800, and 3,400 *m*/*z*, indicating successful binding of Jeffamine to PdPFP and the efficiency of the dialysis method, as well as the presence of PdPFP-Jeff species with 1, 2, 3, or 4 bound Jeffamines in the structure. The absence of free Jeffamine peaks and the presence of peaks corresponding to PdPFP-Jeff with 1, 2, 3, and 4 Jeffamines confirmed the success of the reaction and demonstrated the efficacy of dialysis in purifying the material, dropping the free Jeffamine concentration from 0.65 μM to 0.02 μM in the PdPFP-Jeff compound. The removal of free Jeffamines was essential, as their presence in high concentrations could lead to cytotoxic effects in subsequent biological applications.^33^

**Figure 1:**
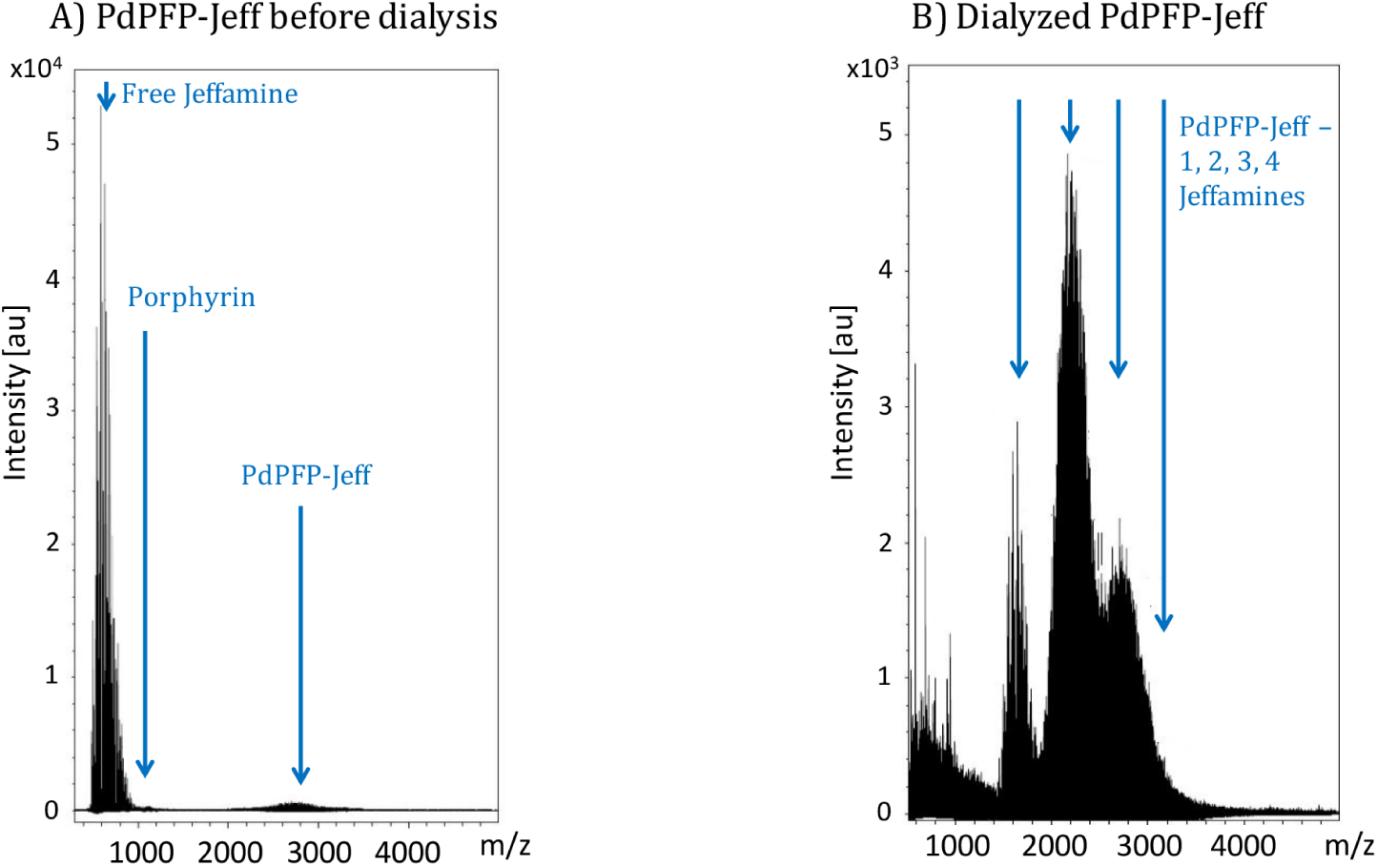
MALDI-TOF mass spectra of (A) nondialyzed and (B) dialyzed PdPFP-Jeff after the synthesis reaction.

### PdPFP-Jeff Chemical Bonding Stability Analysis

The successful inclusion of PdPFP-Jeff into the hydrogels was also confirmed by spectral scanning for different porphyrin concentrations in the hydrogel. As can be observed in Figure 2, the obtained absorbance depends on the concentration of the porphyrin inside the hydrogels, showing a characteristic absorption peak at 420 nm, with its maximum being a concentration-dependent value. This peak is indicative of the excitation wavelength, which is a common value in palladium porphyrin compounds^34^ and signifies the presence of PdPFP-Jeff within the hydrogel structure.

**Figure 2:**
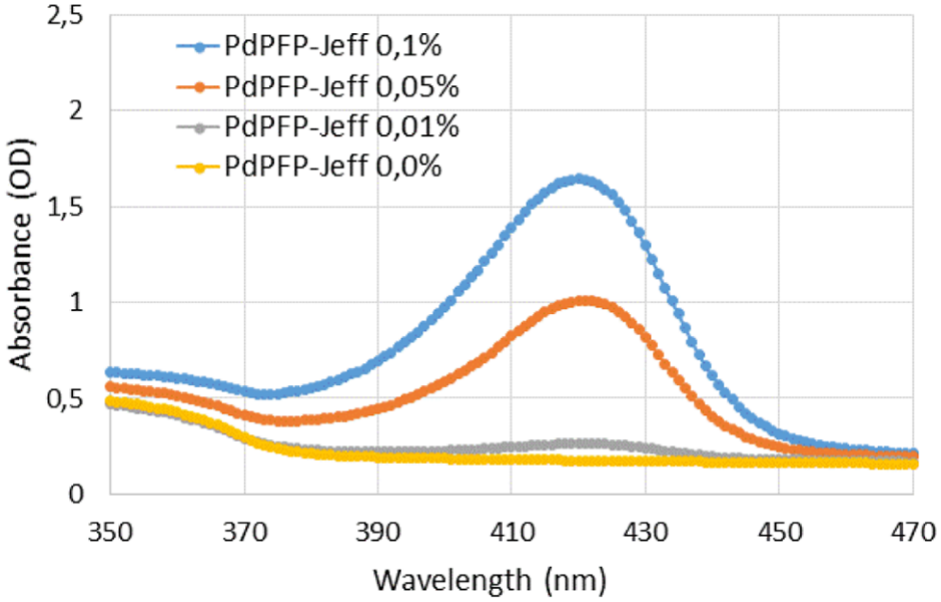
Spectral scanning from 350 to 470 nm of optical densities for different AlgNHS-plasma-derived hydrogel solutions with different PdPFP-Jeff concentrations. The AlgNHS concentration was 0.5 mg/mL. PdPFP-Jeff concentrations were 0%, 0.01%, 0.05%, and 0.1% (w/v). The absorption peak of porphyrin around 420 nm is shown, with peak amplitude proportional to the PdPFP-Jeff concentration.

The PdPFP-Jeff chemical bonding stability was determined by studying the PdPFP release, whereas Figure 3 shows the remaining PdPFP linked to the polymer. Hydrogels with different PdPFP-Jeff and AlgNHS concentrations were incubated in PBS, and supernatants were collected on days 1, 3, 7, and 10 and analyzed using a Synergy HTX Multi-Mode Microplate Spectrophotometer. Optical density at 420 nm was measured to quantify porphyrin release using a calibration curve for concentration determination. The results showed that approximately 10–15% of the incorporated porphyrin was released within the first few days, after which no further porphyrin was released, with the majority remaining covalently bound to the hydrogel matrix, showing a high integration and strong chemical bond (Figure 3). This limited release demonstrates the covalent binding between PdPFP-Jeff and the hydrogel, which is crucial for maintaining its functionality in tissue-engineered constructs. The stability of the chemical bonding was further supported by the minimal release observed after the initial period, indicating that the porphyrin remained securely attached to the hydrogel network. The stable retention of sensing molecules within hydrogel or polymeric matrices is a critical requirement shared across diverse biosensing platforms, as demonstrated in metalloporphyrin-based systems for multimodal analyte detection.^35^ The strong chemical integration of PdPFP-Jeff into the hydrogel matrix ensures that the porphyrin can effectively function as an oxygen biosensor, providing reliable and sustained measurements. It should be noted that over 80% of the porphyrin retained within the hydrogels was chemically bound, and its fluorescence can be measured using established methods^36^ to evaluate hydrogel parameters such as oxygen concentration and stability.

**Figure 3:**
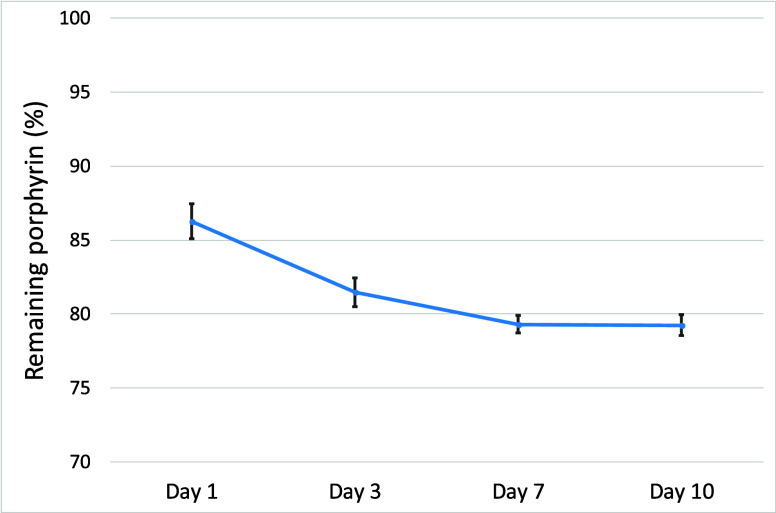
PdPFP remained linked to the fibrin-AlgNHS hydrogel at different times (1–10 days), with 0.5 mg/mL of AlgNHS and 0.1% PdPFP-Jeff concentrations.

### Biocompatibility Analysis of the Oxygen-Sensing Hydrogels

The possible application of PdPFP-Jeff hydrogels in skin and soft tissue engineering was first studied by analyzing the viability and proliferation of hFBs and hKCs in PdPFP-Jeff hydrogel, as described in the Experimental Section “Cell cultures in oxygen sensing hydrogels”.

The first tests involved live/dead assays with hFBs. Figure 4 shows that hFBs embedded in the hydrogels with 0.5 mg/mL of AlgNHS exhibited cell viability similar to that of control hydrogels and significantly better than gels with 1 mg/mL of AlgNHS. These results agree with those reported in the literature, demonstrating lower fibroblast viability as the sodium alginate concentration increases.^25,37^ Higher porphyrin concentrations also resulted in lower cell viability in a dose-dependent manner. The optimal conditions were found to be 0.5% AlgNHS and 0.01% porphyrin, although higher concentrations could be considered for specific experiments. These findings align with previous studies on palladium porphyrins and related metalloporphyrins, which demonstrated increased cell toxicity at higher concentrations in 2D cultures. According to Nyarko et al.,^38^ porphyrin was toxic at concentrations greater than approximately 0.001–0.003 mg/mL. However, depending on the AlgNHS concentration, we observed good cell viability at concentrations in the range of 0.01–0.05% of porphyrin (see Figure 4D,E,G).

**Figure 4:**
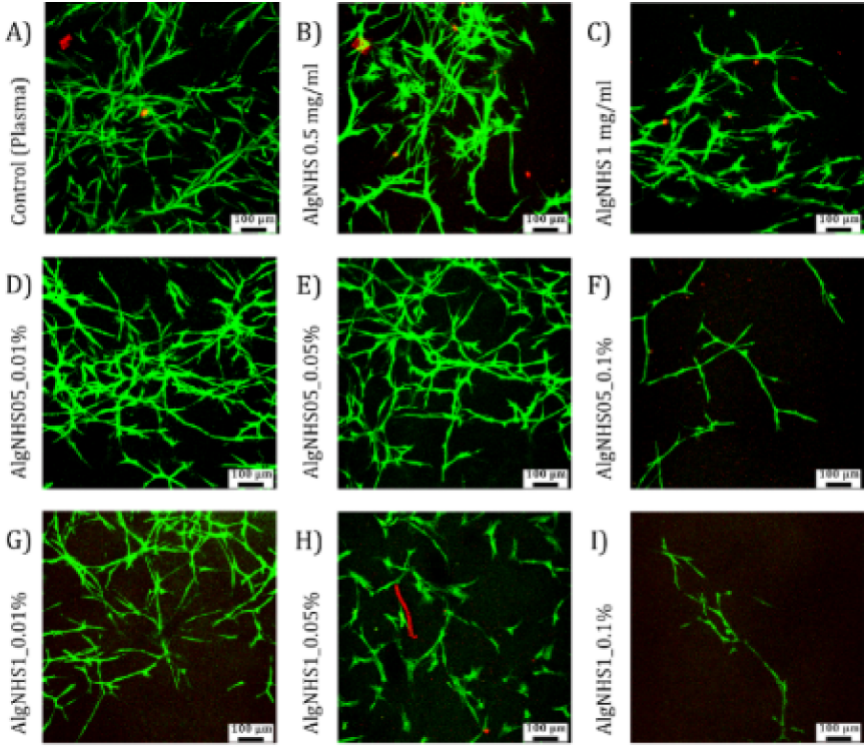
Live/dead assays of hFBs embedded in PdPFP-Jeff (A) plasma-derived fibrin hydrogel; (B) 0.5 mg/mL AlgNHS hydrogel; (C) 1 mg/mL AlgNHS hydrogel; (D–F) PdPFP-Jeff hydrogels with an AlgNHS final concentration of 0.5 mg/mL and PdPFP-Jeff final concentrations of (D) 0.01%, (E) 0.05%, and (F) 0.1% (w/v); (G–I) PdPFP-Jeff hydrogels with an AlgNHS final concentration of 1 mg/mL and PdPFP-Jeff final concentrations of (G) 0.01%, (H) 0.05%, and (I) 0.1% (w/v). Scale bar: 100 μm. The results are representative of three independent experiments.

In similar experiments (Figure 5), hKCs were seeded on the top of PdPFP-Jeff hydrogels with 0.5 mg/mL of AlgNHS (the AlgNHS concentration that showed better results with hFBs). The Live/Dead assay results (Figure 5) showed that hKCs grew similarly well under all the experimental conditions assayed, thus indicating that the synthesized porphyrin PdPFP-Jeff, being chemically bonded in the dermal compartment, had limited effects on the epidermal cells at the surface.

**Figure 5:**
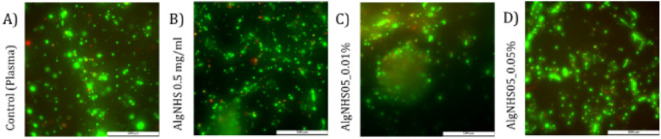
Live/dead assays of hKCs cells grown on the surface of PdPFP-Jeff after 4 days of culture: (A) control plasma-derived fibrin hydrogel; (B) hydrogel with 0.5 mg/mL AlgNHS; and (C, D) PdPFP-Jeff hydrogels with 0.5 mg/mL AlgNHS and PdPFP-Jeff concentrations of (C) 0.01% and (D) 0.05% (w/v). Green spots represent keratinocyte colonies formed from the initially seeded cells. Scale bar: 500 μm. The results are representative of three independent experiments.

### Validation of the Oxygen-Sensing Capacities of the Developed Hydrogels

The subsequent experiments are focused on the fluorescence measurement of porphyrins chemically integrated into PdPFP-Jeff hydrogels under varying oxygen concentrations. The emission spectra of different oxygen-sensing hydrogels made with different concentrations of the PdPFP-Jeff compound were measured from 450 to 750 nm. The results that validate the oxygen-sensing capacity of the developed hydrogels are presented in Figure 6. The fluorescence response was analyzed in PdPFP-Jeff hydrogels with varying total content of PdPFP-Jeff (0.01, 0.05, and 0.1% w/v) at 0.5 mg/mL of AlgNHS (Figure 6). It is important to highlight that the excitation wavelength we used (405 nm) has limited penetration into the hydrogels due to water absorption, so we can assume that the emitted photons are mostly coming from the surface of the hydrogel directly in contact with the air, and thus the oxygen concentration in the upper layer of the hydrogel is the same as in the surrounding environment. In Figure 6 we can see that changes in oxygen levels and corresponding variations in porphyrin intensity were observed as nitrogen was gradually introduced into sealed chambers containing the hydrogels. The resulting oxygen depletion led to a significant increase in fluorescence intensity near 670 nm. Furthermore, fluorescence quenching occurred when oxygen levels returned to atmospheric concentrations, restoring the fluorescence intensity profile to its initial state (reversibility). Additionally, the fluorescence enhancement factor (FeF) was calculated to quantify the degree of quenching in response to oxygen variations. The FeF was obtained at λ_em_ = 672 nm, and it is defined as the ratio between the intensity without oxygen (0%), depleted using nitrogen, and the intensity with oxygen at 21%, in both measurements, after subtracting the autofluorescence of the hydrogel (eq 1).

**Figure 6:**
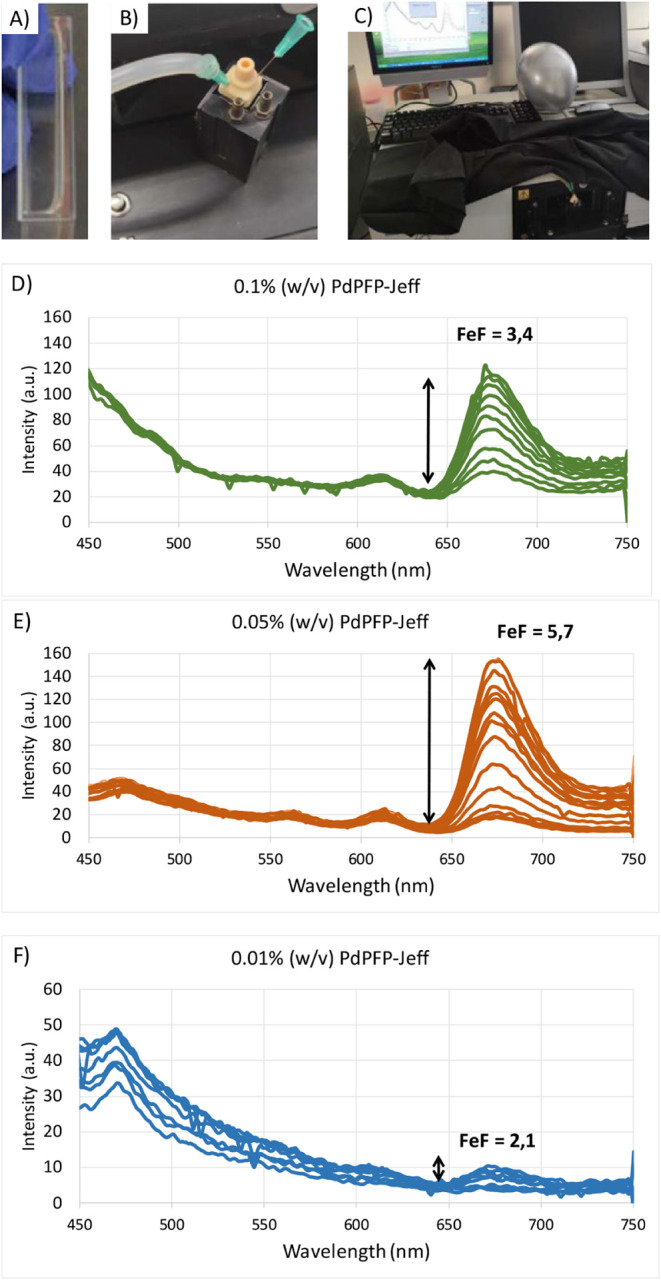
Fluorescence intensity measurements of porphyrin bonded to PdPFP-Jeff hydrogels at different oxygen concentrations from 0% to 21%. Excitation wavelength λ_exc_ = 405 nm. Set-up: (A) 700 μL of hydrogel sample placed in the cuvette; (B) sealed cuvette with needles for gas exchange; (C) cuvette placed in the spectrometer covered by a black blanket. Results: (D) hydrogel with 0.1% (w/v) PdPFP-Jeff; (E) hydrogel with 0.05% (w/v) PdPFP-Jeff; and (F) hydrogel with 0.01% (w/v) PdPFP-Jeff.

The optimal conditions under which the fluorescence intensity of the porphyrin molecule was measurable and yielded a significant difference for different oxygen concentrations on the surface were determined. The experiments confirmed that the fluorescence intensity exhibited a direct correlation with oxygen concentration, supporting the suitability of PdPFP-Jeff as an oxygen sensor (Figure 6). The results showed that at 0.01% porphyrin concentration, the fluorescence intensity was too low for accurate quantification, whereas at 0.05% and 0.1%, significant intensity variations were observed. The FeF values obtained, greater than 2 in these cases, indicate a measurable distinction in fluorescence response with the oxygen concentration of PdPFP-Jeff hydrogels. Results show that at higher porphyrin concentrations, the fluorescence signal is reduced as well, suggesting optimal concentrations below 0.1% (w/v). Once this conclusion was reached, the remaining experiments, including the tests with embedded cells, were carried out at 0.05% PdPFP-Jeff concentration. Additionally, as mentioned before, the reversibility of the system was verified, which is very relevant to potential applications in tissue engineering. This reversibility, together with the high FeF values and dynamic range at 0.05% concentrations, reinforces the robustness of the PdPFP-Jeff hydrogel system as a reliable platform for noninvasive, real-time oxygen sensing in biological environments. Furthermore, the observed reproducibility across repeated cycles of oxygen depletion and reoxygenation suggests that the PdPFP-Jeff system can be employed in long-term monitoring applications, such as in vitro wound healing assays or chronic tissue oxygenation studies.

### Validation of the Oxygen-Sensing Capacities in a Dermis-Like In Vitro Environment

In order to study the impact of the presence of cells on fluorescence intensity and oxygen variability, the next step was to analyze PdPFP-Jeff hydrogels containing cells (hFBs) at a final PdPFP-Jeff concentration of 0.05%, which corresponds to the AlgNHS hydrogels with the highest FeF. The inclusion of human fibroblasts (hFBs) leads to the formation of a dermis-like environment in vitro, enabling the assessment of oxygen dynamics in a more physiologically relevant model. Comparison graphs between PdPFP-Jeff hydrogels with and without hFB cells are shown in Figure 7.

**Figure 7:**
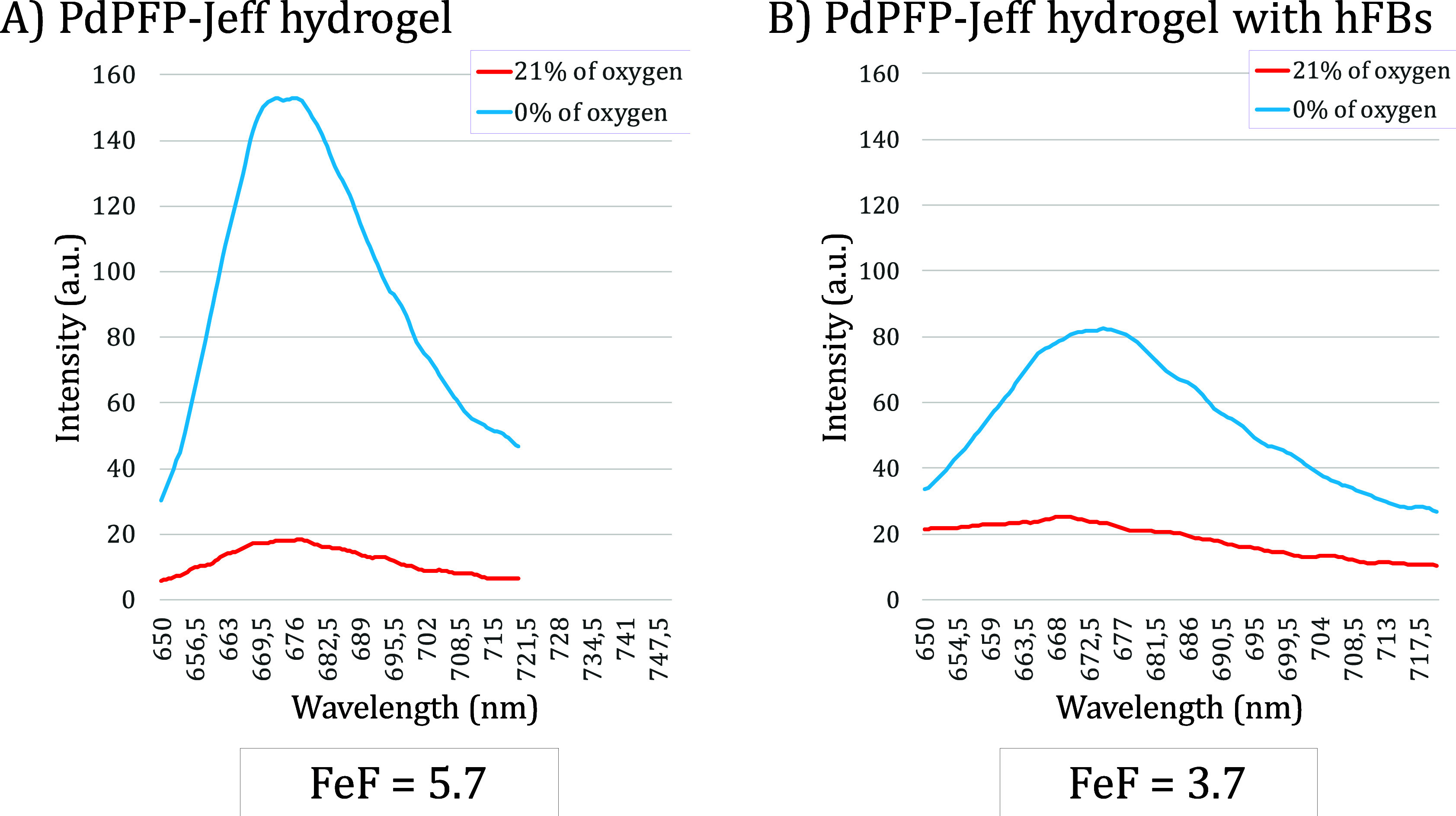
Graphs comparing the fluorescence intensity spectrum and FeF of modified porphyrin in PdPFP-Jeff hydrogels with and without cells (λexc = 405 nm). The PdPFP-Jeff concentration was 0.05% (w/v). The oxygen-absence conditions are represented by the blue lines, while the red lines represent the oxygen concentration of 21%. The FeF is obtained at the maximum emission wavelength (λ_em_ = 672 nm).

As is evident in this instance, the embedded cells significantly reduce the FeF, or the measurable change in intensity, for a given oxygen concentration. This may be because the emitted fluorescence could be absorbed or scattered by the cells, cellular structures, or molecules, which further reduces the fluorescence signal seen in the fluorescence spectrophotometer. In general, scattering of fibroblasts can affect the precision and sensitivity of fluorescence intensity-based assays in cellular studies.^39^ Nonetheless, an FeF = 3.7, greater than 2, represents a noteworthy deviation from atmospheric conditions, rendering this system a quantifiable real-time indicator of the oxygen conditions present in the dermis in vitro. These findings highlight the sensitivity of PdPFP-Jeff hydrogels to oxygen concentration changes. Measuring fluorescence intensity variations is crucial for reliable oxygen sensors. The reduction in FeF with cells highlights the need to consider cellular interactions.

### Validation of PdPFP-Jeff Hydrogels as Stable Matrix Suitable for Skin Tissue Development

In this section, we show that the developed hydrogels provided a viable platform for engineered skin applications. To achieve this, the generated dermo-epidermal equivalents were examined using immunofluorescence assays and hematoxylin and eosin (H&E) staining (Figure 8). The equivalents fabricated using PdPFP-Jeff hydrogels displayed a morphology visually similar to that of the control equivalents lacking PdPFP-Jeff, with both types showing a rough upper surface commonly attributed to the formation of a stratum corneum, which is considered to be a marker of a well-terminally differentiated epidermis. The porphyrin color did not change noticeably over the course of the days, indicating that PdPFP-Jeff was chemically bonded to the matrix and did not affect cell viability, proliferation, or differentiation. H&E staining also revealed that both the cell morphology and structure of the skin equivalents obtained with modified fibrin were similar to those obtained using control, unmodified fibrin. As can be observed in Figure 8, all the analyzed samples indeed had a well-developed stratum corneum, a multilayered epidermis with densely packed keratinocytes, and a dermal compartment sparsely populated with fibroblasts (indicating the generation of well-developed and structured dermo-epidermal equivalents). Additionally, the AlgNHS skin constructs exhibited increased dermal matrix stability, as evidenced by their thicker dermis after 15 days of differentiation.

**Figure 8:**
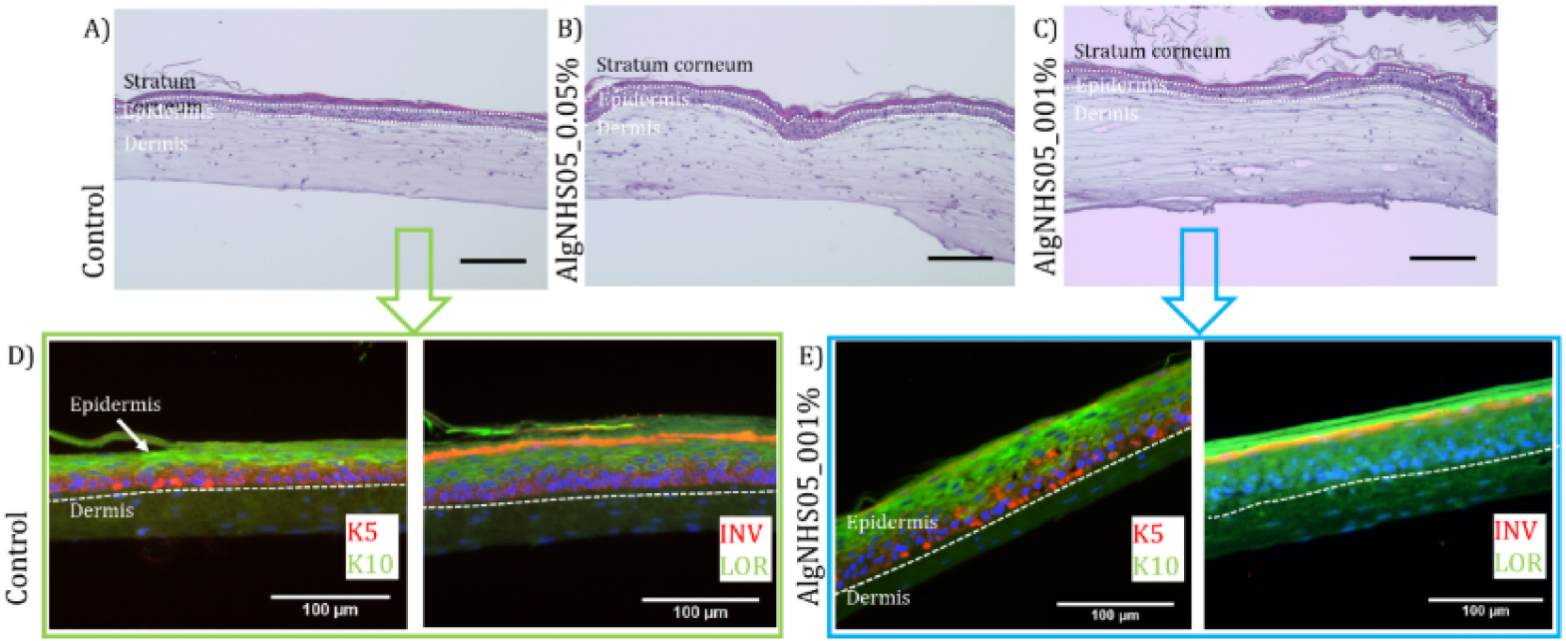
PdPFP-Jeff dermo-epidermal skin equivalents differentiated at the culture liquid–air interface. (A–C) H&E staining of dermo-epidermal skin equivalents from (A) unmodified plasma-derived fibrin hydrogels and PdPFP-Jeff hydrogels (0.5 mg/mL of AlgNHS) with PdPFP-Jeff at final concentrations of (B) 0.01% and (C) 0.05% (w/v). (D–E) Immunostaining of dermal equivalents from (D) control plasma-derived fibrin hydrogel and (E) PdPFP-Jeff hydrogels (0.5 mg/mL of AlgNHS) with PdPFP-Jeff at 0.05% (w/v). (D–E) The samples were analyzed by immunohistochemistry using antibodies against K5 (red, basal epidermal cells) and K10 (green, suprabasal epidermal cells) (left of D and E), and against involucrin (green, suprabasal epidermal cells) and loricrin (red, stratum granulosum, the uppermost layer of living cells beneath the stratum corneum) (right of D and E). DAPI-stained cell nuclei are seen as blue spots. Scale bar: 100 μm.

The dermo-epidermal matrices were also characterized using immunofluorescence to examine the expression of epidermal differentiation markers such as Cytokeratin 5 (K5), Cytokeratin 10 (K10), Involucrin (IVL), and Loricrin (LOR).^40^ The results showed similar expression patterns in PdPFP-Jeff and control fibrin-based 3D skin equivalents (see Figure 8D–E). This analysis also confirmed that the modified porphyrin gels had no effect on keratinocyte differentiation or cell proliferation, making PdPFP-Jeff hydrogels at a final porphyrin concentration of 0.05% (w/v) or below suitable for developing dermo-epidermal matrices with increased mechanical stability and probably also durability, enabling the *in situ* and real-time measurement of oxygen concentration in the engineered skin equivalents.

## Conclusions

This study presents the development and characterization of PdPFP-Jeff-functionalized fibrin-based hydrogels as a platform for noninvasive, real-time oxygen sensing in engineered skin tissues. The chemical modification of palladium porphyrin with Jeffamine (PdPFP-Jeff) enabled its covalent incorporation into AlgNHS-modified plasma-derived fibrin hydrogels through the formation of stable amide bonds. This strategy ensured retention of the porphyrin within the hydrogel matrix without leaching, which is essential for long-term sensing applications. Fluorescence spectroscopy confirmed that PdPFP-Jeff hydrogels exhibited oxygen-dependent fluorescence at 405 nm excitation and 672 nm emission, with the intensity varying inversely with oxygen concentration. The fluorescence enhancement factor (FeF) reached 5.7 at 0.05% (w/v) PdPFP-Jeff in acellular hydrogels and 3.5 in fibroblast-containing samples, demonstrating sufficient sensitivity for oxygen monitoring in cellular environments. The system also showed reversible and reproducible responses across repeated cycles of oxygen depletion and reoxygenation. Importantly, biological characterization showed that PdPFP-Jeff matrices (1.2 mg/mL fibrin, 0.5 mg/mL AlgNHS, and up to 0.05% (w/v) PdPFP-Jeff) supported fibroblast proliferation and keratinocyte differentiation without cytotoxic effects. The resulting dermo-epidermal constructs were morphologically and histologically comparable to control samples, confirming the biocompatibility of the system for skin tissue-engineering applications. In this sense, this work demonstrates the potential of PdPFP-Jeff hydrogels as biosensors for real-time oxygen monitoring in skin equivalents, as well as validates this approach for further advances in smart biomaterials and improvements of in vitro skin models.

These promising results, however, have some limitations that must be acknowledged. The oxygen-sensing characterization was performed using fluorescence intensity measurements only, without complementary lifetime-based analysis, which could provide additional robustness against signal variability. This is relevant, as the reduction in FeF observed in the presence of cells indicates that scattering and absorption by cellular components affect the fluorescence readout, and this effect may become more pronounced in thicker or more complex tissue constructs. Finally, all experiments were conducted in vitro, and the performance of the system under more realistic physiological conditions remains to be evaluated.

In this direction, our current efforts are focused on the integration of phosphorescence lifetime measurements, and multipoint oxygen calibration protocols, which would improve the quantitative accuracy and reliability of the sensing platform, including the use of two-photon fluorescence lifetime imaging (FLIM) techniques for improved penetration. Further work will also focus on in vivo validation using human skin grafts in immunodeficient mice to assess the clinical applicability of PdPFP-Jeff hydrogels, particularly for wound healing monitoring and skin transplant assessment, paving the way for enhanced wound healing and regenerative medicine applications.

## #

Physics Department, University of the Basque Country (UPV/EHU), 48080 Bilbao, Spain, and IKERBASQUE, Basque Foundation for Science, Plaza Euskadi 5, 48009 Bilbao, Spain
